# Continuity-Driven Escalation in Telehealth: Redefining Transitions in Outpatient Care

**DOI:** 10.7759/cureus.109239

**Published:** 2026-05-19

**Authors:** Hussein Jumhour

**Affiliations:** 1 Family Medicine, Private Practice, Paterson, USA

**Keywords:** care escalation, care transitions, clinical decision making, continuity-driven escalation, continuity of care, diagnostic pathways, healthcare delivery, outpatient care, telehealth, virtual care

## Abstract

Current approaches to evaluating telehealth often rely on single-encounter outcomes, which may not reflect how outpatient care unfolds over time. Transitions from virtual to in-person care are frequently interpreted as limitations of remote care rather than as part of clinical decision-making. This editorial introduces the concept of continuity-driven escalation, defined as the clinically guided progression of care across settings within a unified diagnostic pathway. Reframing telehealth within this model shifts evaluation from isolated visits to the broader trajectory of care. Recognizing this distinction may improve the interpretation of telehealth outcomes and support more accurate assessment of care quality.

## Editorial

Telehealth is widely integrated into outpatient clinical practice. However, its effectiveness is often assessed by a simple question: Did the patient require in-person evaluation? When care transitions from telehealth to in-person settings, this is frequently interpreted as a limitation of remote care rather than a necessary step in clinical decision-making. This interpretation becomes more consequential as telehealth is incorporated into routine clinical workflows rather than used as a temporary access strategy [1]. When evaluation is limited to whether care is resolved within a single encounter, the role of telehealth in guiding appropriate clinical progression may be underrecognized.

Interpreting transitions as failures reflects an oversimplification of how outpatient care unfolds. Movement between care settings is not inherently a failure of a specific modality but part of a broader clinical process that extends across encounters. This process can be described as continuity-driven escalation, defined as the clinically guided progression of care across settings within a unified diagnostic pathway. In this context, transitions reflect evolving clinical needs rather than limitations of a specific modality, and escalation represents a response to those evolving needs within a coherent diagnostic process.

The concept aligns with established principles of continuity of care, emphasizing sustained clinical relationships and coordination across encounters as key determinants of outcomes [2]. In this context, escalation is not simply a transfer between modalities but a continuation of care when additional evaluation is required. Uncoordinated transfers that occur without clinical intent or defined follow-through are distinct from continuity-driven escalation, which assumes a shared diagnostic trajectory rather than isolated encounters.

This distinction is important because diagnostic uncertainty is a normal part of clinical care, and errors may arise when evolving clinical information is not effectively incorporated into subsequent decision-making [3]. Failure to recognize this distinction can lead to inaccurate conclusions about telehealth effectiveness, as evaluations that define success as resolution within a single encounter may underestimate the role of telehealth in guiding appropriate next steps in care.

The value of telehealth in this model is not confined to what is resolved during a virtual visit, but also includes the clinical judgment that determines when and where further evaluation is needed. In many cases, escalation from virtual to in-person care may reflect appropriate recognition of diagnostic uncertainty rather than failure of the initial encounter itself. Reframing telehealth within this model shifts the focus from modality to process. The key question is not whether telehealth replaces in-person care, but how it contributes to a broader trajectory of clinical decision-making.

Studies that use single-encounter resolution as a proxy for telehealth effectiveness may not fully capture how outpatient care unfolds over time. A pathway-based perspective may provide a more complete view than an encounter-based approach. Outcome measures that do not distinguish between unresolved care and appropriate escalation may therefore misinterpret both care quality and system performance, particularly in settings where follow-up patterns and transitions are integral to care delivery [4,5]. As hybrid outpatient models continue to expand, evaluation frameworks may need to better account for continuity, coordination, and clinically appropriate escalation across care settings rather than focusing solely on whether care was resolved within a single encounter.
